# Parasite–Host Associations in Pinworms (*Trypanoxyuris* spp.) From Spider Monkeys (*Ateles* spp.)

**DOI:** 10.1002/ajp.70203

**Published:** 2026-08-27

**Authors:** Brenda Solórzano‐García, Andrés Link Ospina, Anthony Di Fiore, Felipe Alfonso‐Cortes, Nathalia Fuentes, Gerardo Pérez‐Ponce de León

**Affiliations:** ^1^ Laboratorio de Parasitología y Medicina de la Conservación ENES‐Mérida, UNAM Mérida Yucatán Mexico; ^2^ Laboratorio de Ecología de Bosques Tropicales y Primatología, Departamento de Ciencias Biológicas Universidad de los Andes Bogotá Colombia; ^3^ Department of Anthropology and Primate Molecular Ecology and Evolution Laboratory The University of Texas at Austin Austin Texas USA; ^4^ Estación de Biodiversidad Tiputini Universidad San Francisco de Quito Quito Ecuador; ^5^ Proyecto Washu Quito Ecuador; ^6^ Fundación Naturaleza y Arte Quito Ecuador

**Keywords:** genetic divergence, incomplete speciation, parasite–host coevolution

## Abstract

Pinworms of primates are highly host‐specific parasites that, in several lineages, are known to share their evolutionary history with that of their hosts. However, for many primate clades, pinworm diversity has been incompletely assessed, leaving some groups of hosts entirely unexplored and large geographic gaps in our knowledge of primate–pinworm associations. In this study, morphological and molecular data (28S rDNA and COI mtDNA) were integrated to assess pinworm diversity across five spider monkey taxa (*Ateles* spp.) from Mexico to Ecuador. Only two species of pinworms were found parasitizing *Ateles* hosts, with morphological traits consistent with *Trypanoxyuris atelis* and *Trypanoxyuris atelophora*. Phylogenetic analyses of 28S sequences corroborated species identities, suggesting minimal intraspecific divergence. In contrast, analysis of COI sequences revealed the existence of distinct lineages that paralleled host taxonomic identity for both pinworm species. Divergence levels remained below thresholds typical of species‐level differentiation within the genus *Trypanoxyuris*, suggesting incomplete speciation following host diversification. These results echo those found in previous studies in other primate taxa and highlight the potential of pinworms as valuable indicators of host evolutionary and biogeographic histories. Our results reveal complex, species‐specific evolutionary trajectories in *Trypanoxyuris* infecting spider monkeys and underscore the importance of comprehensive sampling for investigating parasite–host coevolution in platyrrhines.

## Highlights

- COI data showed distinct pinworms lineages corresponding with *Ateles* taxonomic identity.
- Incomplete speciation may explain diversification in these pinworm species.
- Pinworms could serve as useful proxies for studying primate biogeography and evolution.

## Graphical abstract

Lineages in pinworms from *Ateles* monkeys are highly host‐specific and their diversification patterns reflect host biogeographic and evolutionary histories.

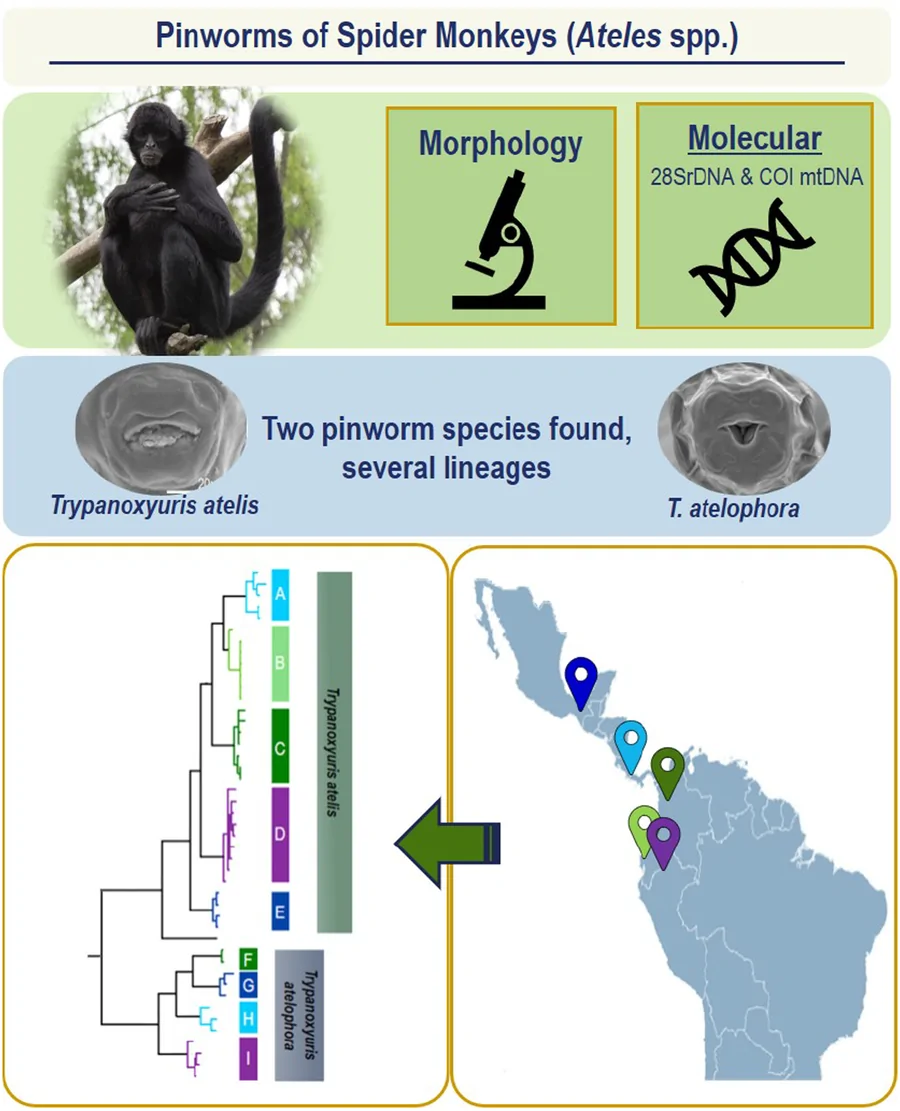

**Abbreviations**
BI: Bayesian Inference
CNHE: Colección Nacional de Helmintos
COI: cytochrome oxidase subunit I
Cytb: cytochrome b
DNA: deoxyribonucleic acid
MCMC: Markov chain Monte Carlo
PCR: polymerase chain reaction
SEM: scanning electron microscopy

## 1 Introduction

Understanding the evolutionary dynamics underlying host–parasite interactions remains a central focus in parasitology, particularly in systems where host specificity strongly influences parasite diversification (Buckingham and Ashby 2022; Hay et al. 2020; Hoberg et al. 1997). Constant coevolutionary feedback to increase fitness and survival generates several patterns of parasite distribution among hosts and differential specificity, often leading to concordance between host and parasite phylogenetic histories (Brooks 1979; Combes 2001; de Vienne et al. 2013).

Host specificity, defined as the degree to which a parasite is restricted to one or a few host species, is a critical determinant of parasite speciation, transmission dynamics, and coevolutionary trajectories (Poulin et al. 2011). Parasites with high host specificity often have narrow ecological niches shaped by intimate ecological, molecular, and physiological associations with their hosts (Poulin 2011). Such relationships may trigger local adaptation processes that result in a high degree of specialization by the parasite, that usually relates to the phylogenetic and biogeographic histories of their hosts (Gandon and Michalakis 2002; Nuismer et al. 2003; Nuismer and Kirkpatrick 2003).

However, patterns of parasite distribution among hosts can also result from evolutionary events other than co‐speciation including host switching, intra‐host speciation or duplication, and failure to speciate in response to host speciation events (Johnson et al. 2003; Page and Charleston 1998). These diversification processes are further complicated by disparities in evolutionary rates of both parasites and their hosts. Parasites, owing to their shorter generation times, higher reproductive rates, and large population sizes, can diverge evolutionarily from one another much more rapidly than their hosts (Bromham et al. 2013; Hafner et al. 1994). This asynchronous tempo may lead to mismatches in phylogenetic tree topologies masking coevolutionary process even in host‐specific parasites (Hafner and Nadler 1990).

Pinworms (Nematoda: Oxyuridae) are parasites with a direct life cycle and no free‐living stages, and they are transmitted through the accidental ingestion of eggs with retro‐infection and autoinfection being common forms of transmission (Anderson 2000). The platyrrhine primates of Mesoamerica and South America are exclusively parasitized by pinworms of the genus *Trypanoxyuris* (Hugot et al. 1996). Where they have been studied, each distinct platyrrhine genus appears to be infected by a particular set of *Trypanoxyuris* species, apparently not shared with any other, more distantly related primate hosts, highlighting the evolutionary specificity of these associations (Hugot 1999; Solórzano‐García et al. 2024).

Spider monkeys (*Ateles* spp., Atelidae) are large‐bodied, arboreal platyrrhines distributed from southern Mexico throughout Central America and into the Amazon basin of Brazil (Campbell 2008). The taxonomic history and species composition of this genus is complex, with experts divided in their assessment of the number of valid species and subspecies, as well as on the phylogenetic position of some clades (Rylands and Mittermeier 2024). One current classification based on molecular data recognizes seven species of spider monkeys (*Ateles geoffroyi, Ateles fusciceps, Ateles hybridus, Ateles paniscus, Ateles marginatus, Ateles belzebuth*, and *Ateles chamek*), with two of them being polytypic (Morales‐Jimenez et al. 2015). It has been proposed that spider monkeys originated in the southern Amazon and experienced a series of diversification events as they expanded into northern South America and, subsequently, dispersed into Central America, reaching as far north as Mexico (Lynch Alfaro et al. 2015; Morales‐Jimenez et al. 2015). However, the sequence of these splits and the timing of dispersal events remain uncertain due to persistent ambiguities in the phylogenetic relationships among spider monkeys.

To date, only two species of pinworms have been documented as parasites of spider monkeys, *Trypanoxyuris atelis* and *Trypanoxyuris atelophora* (Hasegawa et al. 2004; Hugot 1985; Solórzano‐García et al. 2015). However, research on pinworms diversity in spider monkeys has focused primarily on *A. geoffroyi* populations occurring in Mexico and Costa Rica, leaving much of the genus's taxonomic and geographic range unexplored.

In this study, morphological and molecular data were integrated to assess pinworm diversity across five taxa of spider monkeys including four species *(A. geoffroyi, A. fusciceps, A. hybridus, A. belzebuth*) and two subspecies (*A.g. vellerosus, A.g. frontatus*) from across a broad swath of the genus' geographic distribution (Mexico, Costa Rica, Colombia, and Ecuador). Phylogenetic and genealogical relationships of the parasites were inferred to identify distinct evolutionary units. Genetic divergence was quantified among both pinworm clades and *Ateles* hosts to compare evolutionary rates between parasites and primates. Finally, the observed patterns of pinworm lineage divergence were examined in relation to host diversification to discuss whether pinworm genetic divergences can provide insights into the biogeographical and evolutionary history of spider monkeys.

## 2 Methods

### 2.1 Ethical Statement

This study was carried out entirely through noninvasive sampling under the permits MAE‐DNB‐CM‐2019‐0117 and CO48775, with no direct contact with individual animals; adhered to the American Society of Primatologists (ASP) Principles for the Ethical Treatment of Non‐Human Primates, and the ASP Code of Best Practices for Field Primatology.

### 2.2 Collection of Specimens

*Trypanoxyuris* specimens were collected from free‐living populations of three species of spider monkeys in South America. Pinworms were recovered from: five pinworm‐positive individuals of *A. hybridus* in San Juan de Carare, Colombia; four pinworm‐positive individuals of *A. fusciceps* in Guayaquil, Ecuador; and six pinworm‐positive individuals of *A. belzebuth* from the Tiputini Biodiversity Station in Orellana, Ecuador. Adult pinworms were recovered from the feces of these primates in situ and fixed in 100% ethanol for further morphological and molecular analysis.

To establish a linkage between morphological features and DNA sequences, some pinworm specimens of each spider monkey species were cut in half with the anterior portion used for morphological examination and the remaining posterior portion used for DNA extraction. Full body photomicrographs were taken using a Leica DM 1000 LED microscope (Leica, Germany) for all the specimens before performing the cuts. Additionally, pinworm specimens previously collected from *A. geoffroyi vellerosus* and *A.g. frontatus* from Mexico and Costa Rica, respectively (Solórzano‐García et al. 2019), were included in the subsequent molecular and morphological analysis.

### 2.3 Morphological Examinations

Worms collected from each host species and subspecies were cleared with a 50:50 alcohol–glycerol solution, observed, and measured using a Leica DM 1000 LED microscope. Measurements of complete pinworm species are presented in micrometers unless otherwise stated. Enface view observations of each pinworm specimen were made following the technique proposed by Solórzano‐García et al. (2024). The saved anterior portions of the cut specimens were processed for scanning electron microscopy (SEM) by dehydration through a graded ethanol series and then critical point dried with carbon dioxide. The specimens were mounted on metal stubs with carbon adhesive, and then gold‐coated and examined in a 15 kV Hitachi Stereoscan Model SU1510. Complete pinworm specimens from *A. fusciceps, A. hybridus*, and *A. belzebuth* were deposited in the Colección Nacional de Helmintos, Instituto de Biología, Universidad Nacional Autónoma de México (UNAM) (CNHE: 12579–12582).

### 2.4 DNA Extraction and Amplification

Posterior portions of cut pinworms were individually digested overnight at 56°C in a solution containing 10 mM Tris‐HCl (pH 7.6), 20 mM NaCl, 100 mM EDTA (pH 8.0), 1% Sarkosyl, and 0.1 mg/mL proteinase K. DNA was extracted from the supernatant using the DNAzol reagent (Molecular Research Center, Cincinnati, OH) according to the manufacturer's instructions. A fragment of the mitochondrial cytochrome c oxidase subunit 1 gene (COI) and a region of the large subunit of the nuclear ribosomal gene (28S) were amplified by PCR. The primers employed for amplification and sequencing of the 28S locus included forward 391: 5′‐AGCGGAGGAAAAGAAACTAA‐3′, and reverse 501: 5′‐TCGGAAGGAACCAGCTACTA‐3′ (Nadler and Hudspeth 1998); the PCR conditions included initial denaturation at 94°C for 3 min, followed by 33 cycles at 94°C for 30 s, 54°C for 30 s, 72°C for 1 min, and a post‐amplification extension for 7 min at 72°C. For the COI locus, primers were COIintF: 5′‐TGATTGGTGGTTTTGGTAA‐3′, and TryCoxR: 5′‐ AACCAACTAAAAACCTTAATMC‐3′ (Casiraghi et al. 2001; Solórzano‐García et al. 2015), and the PCR conditions included initial denaturation at 94°C for 3 min, followed by 35 cycles at 94°C for 30 s, 45°C for 45 s, 72°C for 1 min, and a post‐amplification extension for 7 min at 72°C. PCR products for both loci were treated with Exo‐SAP‐IT (Thermo Scientific) and sequenced via dye‐terminator Sanger sequencing at the sequencing facility of the Instituto de Biología, UNAM. Sequences obtained in this study were deposited in GenBank (PZ718919–42, PZ7122076–103, Table S1).

### 2.5 Phylogenetic and Genealogical Inference

In order to cover as much host diversity as possible, we generated a set of data for phylogenetic analysis by combining the sequences generated here for novel specimens with published sequences of *T. atelis* and *T. atelophora* from Mexican *A.g. vellerosus* and from Costa Rican *A.g. frontatus* (Solórzano‐García et al. 2019), available from the NCBI GenBank database, as well as those reported from captive populations of *A. geoffroyi* and *A. belzebuth* in Japan (Hasegawa et al. 2012). The final data set included DNA sequence data for both pinworm species recovered from five spider monkey taxa covering a wide range of the host distribution spanning from Mexico to Ecuador, as well as sequences from other *Trypanoxyuris* species from different Platyrrhine hosts (Table S1). *Oxyuris equi*, a parasite of domestic horses, was used as outgroup in all phylogenetic analysis performed.

Alignments of both genes were performed using MUSCLE (Edgar 2004) through the EMBL‐EBI web interface (Madeira et al. 2024). The final alignment of the 28S gene consisted of 51 sequences of 1139 bp, and the COI alignment included 50 sequences of 707 bp. The best substitution model for the 28S (GTR + G) and COI (TN93 + I + G) genes was inferred using MEGA v. 11 (Tamura et al. 2021) under the AICc. Phylogenetic analyses were conducted independently for each gene through Bayesian Inference (BI) using the software MrBayes v.3.2.6 (Ronquist and Huelsenbeck 2003) and the CIPRES Science Gateway (Miller et al. 2010). The BI analyses consisted of two simultaneous MCMC runs of four million generations each, using a heating parameter value of 0.2, with a “burn‐in” of 25% and sampling trees every 4000 generations to generate the posterior distribution. A 50% majority‐rule consensus tree was then constructed from the set of sampled post burn‐in trees. Genealogical relationships among haplotypes from both *Trypanoxyuris* species infecting spider monkeys were explored through the construction of minimum spanning haplotype networks using PopArtv.1.7 (Leigh and Bryant 2015) with epsilon set to 0.

### 2.6 Genetic Divergence Analyses

Genetic p‐distance between *Trypanoxyuris* linages from different *Ateles* hosts was calculated for both genes (28S and COI) using the software MEGA v.11 using the pairwise‐deletion option. Additionally, to compare rates of evolution between host and parasite, a MUSCLE alignment with published sequences of the cytochrome b gene (cytb) was built for nine taxa of spider monkeys (including the seven recognized *Ateles* species and the corresponding subspecies considered in this study) (Table S2). The COI and cytb genes have been reported to show a highly similar rates of evolution in primates (Andrews and Easteal 2000); since COI data are not available for all spider monkey, using cytb allowed for a complete coverage of the range of *Ateles* taxa included here. A Spearman's correlation test between host and parasite was applied to evaluate whether more divergent hosts also harbored more divergent parasite lineages. Finally, a Wilcoxon signed rank test was performed to compare the degree of genetic divergence between pinworm samples to that of between their spider monkey hosts. All statistical tests were performed with using the {stats} for the statistical programming software, R version 4.2.2 (R Core Team 2022) and visualizations were run using the package ggplot2 (Wickham 2016).

## 3 Results

A total of 81 pinworms were examined; *Trypanoxyuris atelis* and *T. atelophora* were the only species occurring in the sampled hosts. Multiple individual specimens of *T. atelis* were recovered from each of three newly screened species of *Ateles*: *A. hybridus* (*N* = 15), *A. belzebuth* (*N* = 26), and *A. fusciceps* (*N* = 20). Specimens of *T. atelophora* were also recovered from both *A. hybridus* (*N* = 2) and *A. belzebuth* (*N* = 10), but no individuals of this pinworm species were observed in any of the *A. fusciceps* samples. All recovered specimens of both pinworm species were females. Additionally, new morphometric data are presented for eight specimens of both pinworm species from *A.g. frontatus* (Tables 1 and 2).

**Table 1:** Measurements of adult female *Trypanoxyuris atelis* from spider monkeys (*Ateles* spp.) from Middle and South America and its comparison with previous descriptions.

Host	*A. geoffroyi vellerosus*	*A. geoffoyi frontatus*	*A. fusciceps*	*A. hybridus*	*A. belzebuth*	*A. geoffroyi*
Origin	Mexico	Costa Rica	Colombia	Colombia	Ecuador	Japan
Reference	Solórzano‐García et al. (2015)	This study	This study	This study	This study	Hasegawa et al. (2004)
*N*	25	4	11	8	15	11
Length (mm)	3.1–6.5 (4.6)	4.3–5.5 (4.9)	3.7–5.8 (4.7)	4.5–5.5 (5.1)	3.8–6.1 (5.2)	4.3–5.3 (4.7)
Width in mid body	213.7–491.4 (325.8)	295.2–466.9 (375.0)	281.5–446.6 (338.8)	359.5–466.9 (411.1)	269.5–489.9 (392.0)	267–351 (323.8)
Nerve ring	164.4–273 (203.9)	214.1–497.1 (299.2)	21,102–321.7 (265.4)	252.2–307.8 (276.6)	192.1–497.1 (303.2)	176–205 (188.9)
Esophagus length	776.3–946.4 (863.1)	740.7–1109.3 (926.2)	225.5–908.6 (756.5)	928.5–1018.4 (977.6)	413.1–1175.2 (961.5)	743–864 (804)
Esophagus corpus length	652.8–806.4 (734.6)	657.6–965.1 (793.6)	615.1–848.6 (718.0)	796.3–898.7 (852.4)	373.1–1033.1 (848.3)	605–718 (661.9)
Esophagus width at middle	30.5–54.6 (40.7)	33.5–51.2 (43.1)	31.7–55.6 (42.2)	44.9–51.8 (47.9)	21.9–48.1 (41.6)	37–45 (41.6)
Bulb length	105.3–154.7 (131.1)	110.4–144.2 (127.1)	83.1–135.8 (109.7)	110.4–132.3 (125.2)	40–187.3 (124.5)	122–128 (125.2)
Bulb width	88.8–156 (121.3)	101.9–141.5 (126.7)	83.2–123.6 (99.9)	119.9–140.8 (130.9)	91.1–146.5 (128.7)	112–128 (119.8)
Excretory pore – anterior end	609.2–1372 (974.4)	557.2–1180.4 (989.4)	469.2–1227.8 (758.3)	976.8–1169.3 (1092.2)	806.3–1301.1 (1120.3)	784–1050 (925.6)
Vulva – anterior end (mm)	1.1–1.9 (1.6)	0.9–1.7 (1.4)	0.90–2.0 (1.2)	1.4–1.7 (1.6)	0.9–2.2 (1.4)	1.3–1.7 (1.4)
Tail length (mm)	420.3–1010 (718.3)	637–1231.5 (866.8)		1119.5–1231.5 (1174.7)	1127.1–1641 (1324.7)	950–1240 (1090)
Eggs length	32.5–43.8 (39.2)	36.3–43.0 (38.5)	32.6–40.7 (36.8)	39.1–42.9 (41.6)	34.5–44.6 (40.2)	40–46 (42.1)
Eggs width	16.3–26.8 (20.3)	19.3–21.6 (20.4)	17.9–22.7 (19.6)	18.8–22.0 (20.8)	16.8–23.1 (20.5)	21–24 (22.3)

*Note:* Measurements are presented in micrometers (μm) unless otherwise noted. Range followed by mean values in parentheses.

**Table 2:** Measurements of adult female *Trypanoxyuris atelophora* from spider monkeys (*Ateles* spp.) from Middle and South America and its comparison with previous descriptions.

Host	*A. geoffroyi vellerosus*	*A. geoffoyi frontatus*	*A. belzebuth*	*A. geoffroyi*	*A. geoffroyi*
Origin	Mexico	Costa Rica	Ecuador	Japan	Panamá
Reference	Solórzano‐García et al. (2015)	This study	This study	Hasegawa et al. (2004)	Kreis (1932)
*N*	17	4	10	1	2
Length (mm)	2.7–7.7 (4.8)	3.48–6.81 (4.7)	4.3–6.8 (5.1)	3.88	7.4–8.8 (8.2)
Width in mid body	284.1–634 (426.7)	347.9–558.3 (423.9)	326.5–559.5 (416.8)	280	
Nerve ring	137.8–244.7 (187.2)	190.8–220.5 (437.4)	238.4–294.6 (257.3)	163	
Esophagus length	361.4–522.9 (420.7)	399.1–507.5 (437.4)	335.3–479.5 (411.9)	422	
Esophagus corpus length	80.7–161.6 (119.8)	125.6–161.6 (137.5)	270.7–335.3 (301.1)	157	
Esophagus width at middle	35.9–66.3 (47.6)	35.9–54.8 (43.2)	42.1–44.4 (43.3)	48	
Median bulb length	47.6–93.4 (69.1)	58.5–93.4 (71.4)	43.8–72.6 (60.2)	61	65–80 (72.5)
Median bulb width	51.4–107.2 (85.8)	64.2–105.9 (82.1)	58.9–90.1 (71.8)	74	95–115 (105)
Isthmus length	37.6–87.2 (66.6)	52.8–87.2 (66.1)	55.6–77.2 (64.2)	48	
Bulb length	112.2–201.3 (158.9)	139.7–197.5 (165.1)	116.8–171.5 (147.8)	138	150–160 (155)
Bulb width	83.2–154.1 (120.6)	83.5–143.2 (115.1)	65.4–144.3 (96.7)	99	135–140 (137.5)
Excretory pore – anterior end	696.2–1395 (951.4)	786.5–1136 (951.5)	590.2–732.4 (638.4)	582	
Vulva – anterior end (mm)	1.2–2.7 (1.7)	1.5–1.98 (1.67)	1.1–2.3 (1.6)	1.1	
Tail length (mm)	1.14–2.71 (1.64)	0.93–1.08 (1.03)	1.3–2.1 (1.6)	0.74	
Eggs length	34.1–50.5 (45.5)	43.5–46.5 (44.8)	44.9–45.7 (45.3)	—	37.5–45 (41.6)
Eggs width	22.2–28.7 (24.8)	22.7–25.8 (23.8)	22.3–22.7 (22.5)	—	15–25 (19.4)

*Note:* Measurements are presented in micrometers (μm) unless otherwise noted. Range followed by mean values in parentheses.

### 3.1 Morphological Features

**Figure 1:**
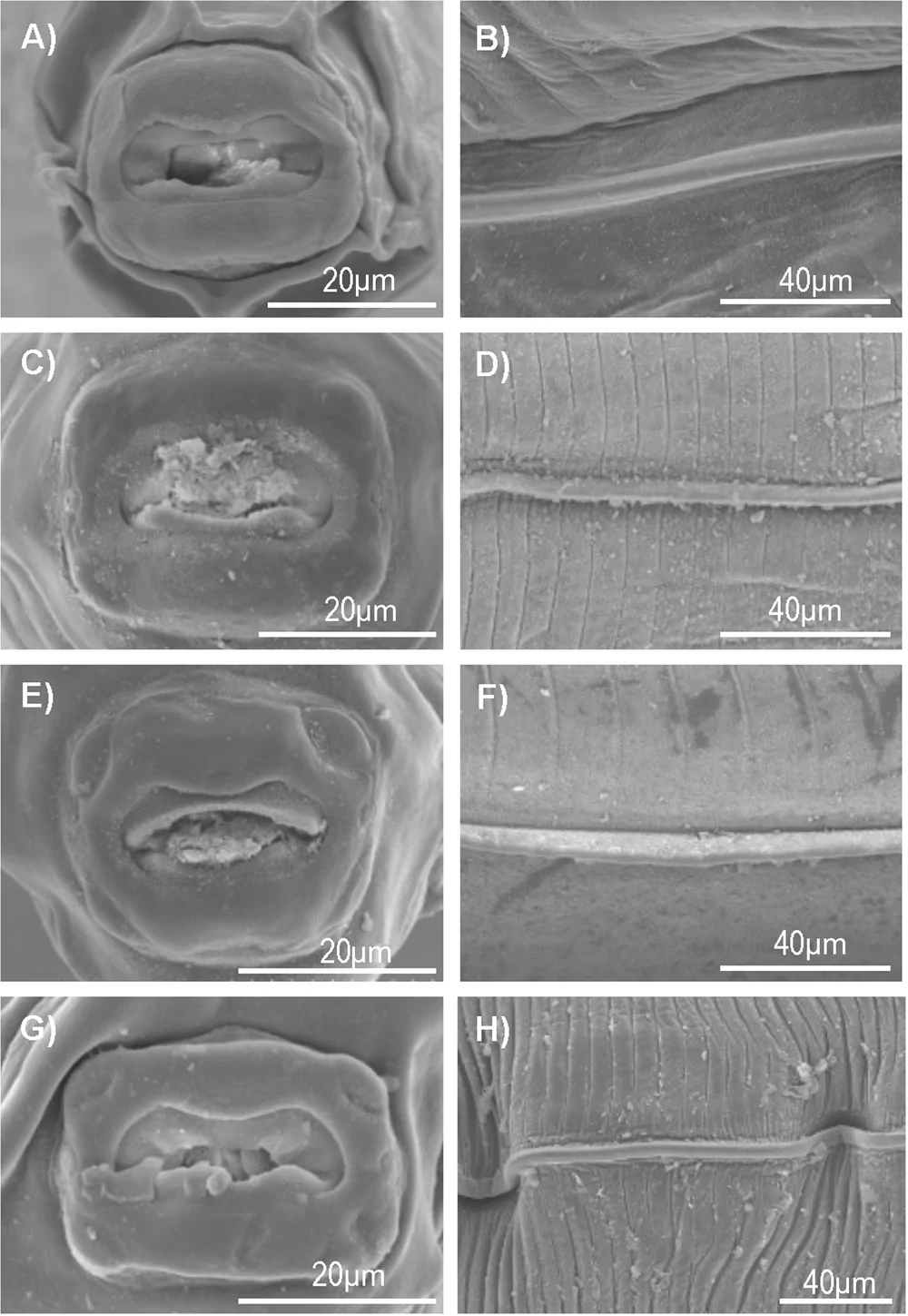
Photographs from scanning electron microscopy of *Trypanoxyuris atelis* from different spider monkey hosts. Left = apical view; right = lateral alae. Pinworm specimens from: (A and B) *Ateles geoffroyi*; (C and D) *A. hybridus*; (E and F) *A. fusciceps*; (G and H) *A. belzebuth*. Differences in the shape of the cephalic plate are artifacts caused by fixation procedures.

Both species of *Trypanoxyuris* from the five *Ateles* species coincide with diagnostic traits of the original species descriptions and more recent redescriptions (Hasegawa et al. 2004; Solórzano‐García et al. 2015). Females of *T. atelis* are characterized by having a cephalic plate with four cephalic papillae at each extreme, and an oral aperture transversally elongate; two laterally elongated lips (one dorsal and one ventral); single crested lateral alae (Figure 1); and an esophagus with only a posterior bulb.

**Figure 2:**
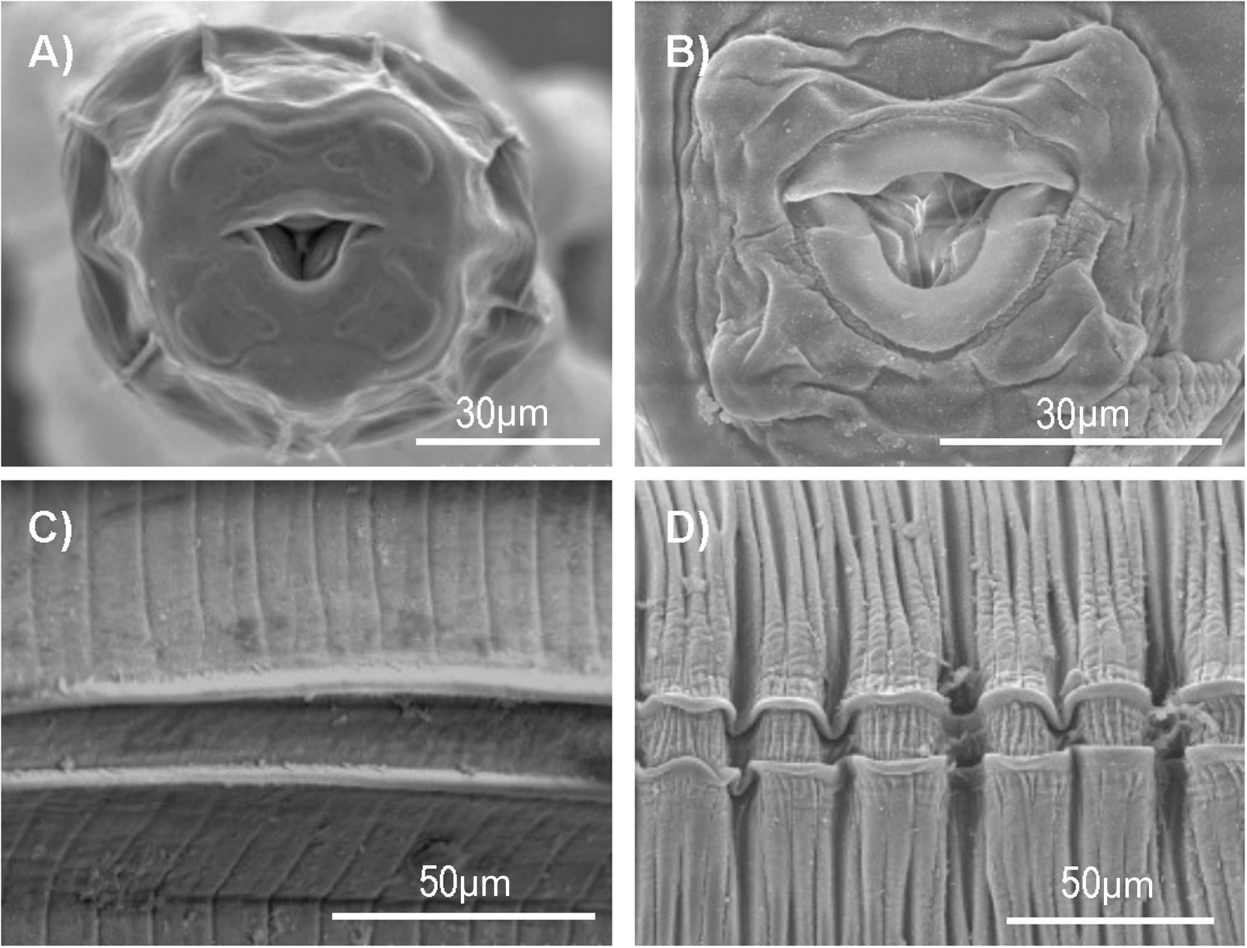
Photographs from scanning electron microscopy of *Trypanoxyuris atelophora* from different spider monkey hosts. Top = apical view; bottom = lateral alae. Pinworm specimens from: (A and C) *Ateles geoffroyi*; (B and D) *A. belzebuth*. Cuticle wrinkles observed in (D) are caused by fixation procedures.

Female *Trypanoxuyris atelophora* are characterized by possessing a quadrangular cephalic plate with four cephalic papillae at each extreme; one dorsal lip and one deeply concave ventral lip forming a triangular oral aperture; double crested lateral alae (Figure 2); and an esophagus with median and posterior bulb separated by an isthmus. Measurements of the pinworm specimens from different hosts fall within the reported range for each of these species (Tables 1 and 2). No particular features, sizes, or proportions were observed that allowed differentiation between specimens from different hosts in either *Trypanoxyuris* species, other than differences resulting from fixation procedures, which are not systematically informative (Figures 1 and 2). Only *T. atelophora* from *A. belzebuth* shows noticeably wider lips than the specimens recovered from the other hosts (Figure 2B).

### 3.2 Phylogeny and Genetic Divergence

**Figure 3:**
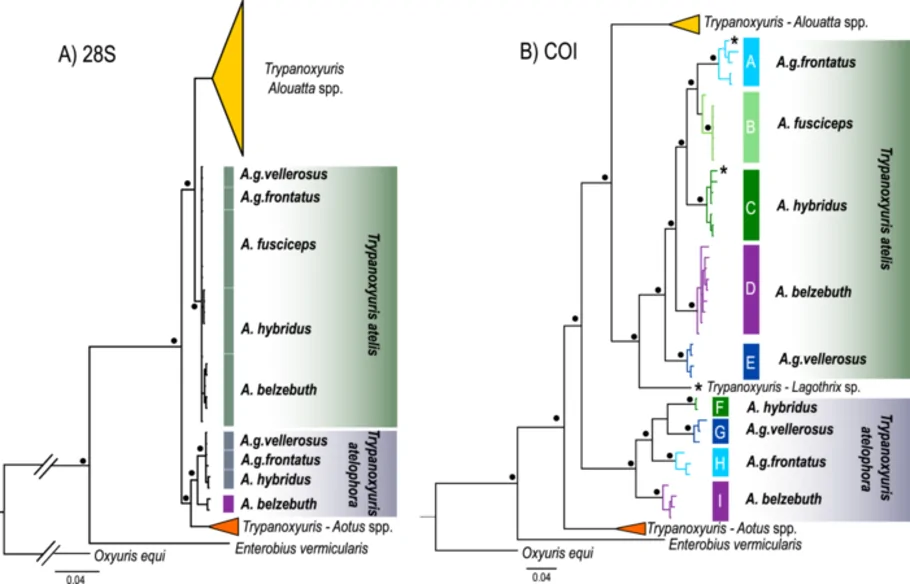
Bayesian phylogenetic tree of *Trypanoxyuris* spp. inferred with (A) 28S and (B) COI genes. Dots at the nodes represent posterior probability values higher than 0.95. Each color and letter indicate a clade of *Trypanoxyuris*. GenBank accessions numbers of sequences are shown in Table S1. *Pinworm sequences obtained from hosts reared in zoos.

Phylogenetic assessment of the 28S gene placed all examined pinworms within expected clades, corroborating the morphological designation of specimens as either *T. atelis* and *T. atelophora*; no subclades were observed in either host species or subspecies (Figure 3). The 28S sequences were almost identical within both *Trypanoxyuris* species, regardless of host identity, showing a mean intraspecific genetic divergence of 0.1% in *T. atelis* and 1.1% in *T. atelophora* (Table 3). Only *T. atelophora* from *A. belzebuth* showed a notably higher level of genetic differentiation (1.6%), and these samples formed their own, separated clade in the phylogenetic tree (Figure 3). In phylogenetic analysis with other species of *Trypanoxyuris*, the two species of pinworm parasitizing spider monkeys were not recovered as sister taxa; rather, *T. atelis* was inferred as the sister taxa to *Trypanoxyuris* species from howler monkeys (*Alouatta*), and *T. atelophora* formed a major clade with *Trypanoxyuris* from night monkeys (*Aotus*) (Figure 3).

**Table 3:** Average genetic divergence between *Trypanoxyuris* lineages, expressed as percentage.

	*Trypanoxyuris atelis* linages					*Trypanoxyuris atelophora* linages				
	A (A.g.f)	B (A.fus)	C (A.hyb)	D (A.bel)	E (A.g.v)	F (A.hyb)	G (A.g.v)	H (A.g.f)	I (A.bel)	*Tryp. minutus* (Al.pall)
Linage A	**2.0**	0.03	0.1	0.1	0.0	3.9	3.6	3.6	3.9	3.8
Linage B	4.4	**0.0**	0.1	0.1	0.03	3.9	3.6	3.7	3.9	3.8
Linage C	5.6	4.3	**1.0**	0.1	0.1	3.9	3.6	3.7	3.9	3.9
Linage D	6.6	5.5	5.4	**2.0**	0.1	3.9	3.6	3.7	3.9	3.8
Linage E	7.1	5.7	6.2	5.4	**1.0**	3.9	3.6	3.6	3.9	3.8
Linage F	13.5	12.7	13.3	13.6	12.3	**0.0**	0.2	0.3	1.6	6.1
Linage G	13.6	12.6	13.1	12.9	12.9	6.5	**1.0**	0.1	1.6	5.9
Linage H	13.9	11.4	12.4	12.9	12.1	7.2	7.0	**2.0**	1.8	5.9
Linage I	13.1	12.5	12.0	13.0	11.9	7.7	8.1	6.7	**1.0**	5.9
*Trypanoxyuris minutus*	9.9	9.8	10.2	9.9	9.2	12.2	13.0	11.6	10.7	**1.0**

*Note:* Above diagonal 28S p‐distance; below diagonal COI values. Shaded values denote intraspecific lineages. Bold values on the diagonal correspond to COI genetic divergence within clades. Host species indicated in parentheses. Hosts: A.g.f = *Ateles geoffroyi frontatus*; A.g.v = *Ateles geoffroyi vellerosus*; A.fus = *Ateles fusciceps*; A.hyb = *Ateles hybridus*; A.bel = *Ateles belzebuth*; Al.pall = *Alouatta palliata*.

In contrast to 28S, the phylogenetic reconstruction with COI sequences revealed the presence of reciprocally monophyletic clades in both *T. atelis* and *T. atelophora* that conformed with host species and subspecies identity (Figure 3). Mean genetic divergence among these clades was 5.6% (4.3%–7.1%) within *T. atelis* and 7.2% (6.5%–8.1%) within *T. atelophora* (Table 3). The lineages of these two parasite's species occurring in each *A. geoffroyi* subspecies were not recovered as monophyletic groups. Within the *T. atelis* group, the lineage occurring in *A.g. vellerosus* (lineage E) was placed at a basal position, whereas within *T. atelophora*, pinworms from *A. belzebuth* (lineage I) were reconstructed as basal and those from *A.g. vellerosus* (lineage G) were recovered as comprising a sister clade to those from *A. hybridus* (lineage F) (Figure 3). These lineages were also evident in the genealogical network of the recovered haplotypes, which reveal the high level of genetic variation between haplotype groups of pinworms from different hosts (Figure S1).

**Figure 4:**
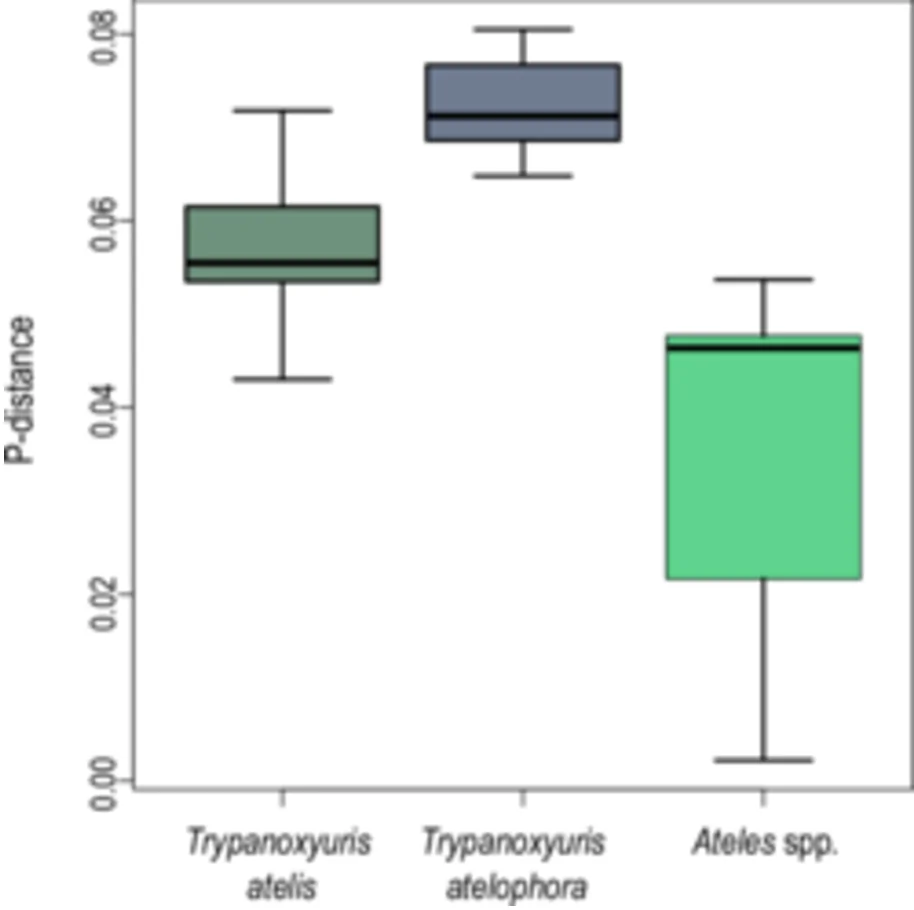
Mitochondrial genetic divergence in the two species of pinworms and its spider monkey hosts. Higher rates of evolution were observed in each parasite species compared to the host (*T. atelis* vs. *Ateles*: *v* = 50, *p* = 0.02; *T. atelophora* vs. *Ateles*: *v* = 21, *p* = 0.03). Also, *T. atelophora* showed higher differentiation than *T. atelis* (*w* = 56, *p* = 0.008).

Comparison of the levels of genetic divergence in mitochondrial genes among hosts and parasites revealed a higher rate of evolutionary change among pinworms lineages compared to their *Ateles* hosts (Figure 4). Furthermore, *T. atelophora* showed higher levels of differentiation than *T. atelis*. Nonetheless, a Spearman test showed no correlation between the genetic divergence in parasite and host lineages for either pinworm species (*T. atelis*: *ρ* = −0.328, *p* = 0.35; *T. atelophora*: *ρ* = 0.28, *p* = 1).

## 4 Discussion

The intimate relationships between pinworms and their primate hosts have long been recognized, particularly due to their high host specificity and the congruence of their evolutionary and genetic patterns (Brooks and Glen 1982; Hugot 1999; Solórzano‐García et al. 2021; Sorci et al. 1997, 2003). The molecular, phylogenetic, and morphological data show that the spider monkey taxa included in this study are parasitized by only two pinworm species: *T. atelis* and *T. atelophora*. This observation is supported by DNA sequence data from the 28S gene, which recovered reciprocal monophyletic groups of each species; by the minimal intraclade variation in this gene; and by the absence of consistent morphological differences among specimens from different species/subspecies of spider monkeys. Further intensive specimen collection is needed to recover male specimens and corroborate the morphological stasis shown by females.

The presence of only these two pinworms in *Ateles* spp. agrees with previous reports (Hugot 1985; Solórzano‐García et al. 2015), suggesting parasite specificity at the host genus level. This pattern is also consistent with previous studies showing that the degree of host specificity in pinworms varies across taxa, with some species occurring in congeneric hosts, whereas others are restricted to single host species (Hugot et al. 1994; Solórzano‐García et al. 2016, 2024, 2020). No specimens of *T. atelophora* were recovered from *A. fusciceps*; more extensive sampling, including populations from across the host's geographic range, is needed to determine whether this host is parasitized exclusively by a single pinworm species.

The phylogenetic results also show that *T. atelis* and *T. atelophora* are not closely related species, a pattern also recovered in prior analyses (Solórzano‐García et al. 2015, 2024). This pattern, however, differs from that observed in other species of *Trypanoxyuris*, where species occurring in the same primate genus (e.g., *Alouatta* and *Aotus*) form monophyletic groups (Solórzano‐García et al. 2024). The lack of monophyletic origin of pinworms from spider monkeys suggests, specifically, that *T. atelis* may have been acquired via host switching event, potentially from howler monkeys (*Alouatta*), given the close phylogenetic association of these two groups of pinworms. Broader sampling across the members of Atelidae remains essential to test this hypothesis and to clarify the evolutionary history of their pinworms.

In contrast to the low variability of the 28S gene, mitochondrial COI assessment revealed well‐defined lineages within each pinworm species that correspond closely with host taxonomic identity, even at the host subspecies level. Previous population genetic studies of *Trypanoxyuris* infecting spider and howler monkeys across Mexico and Central America have also provided clear evidence that pinworms track intraspecific phylogeny and taxonomy within both of these primates (Solórzano‐García et al. 2019). The phylogenetic relationships recovered here for these *Trypanoxyuris* lineages closely resemble those of their hosts (Morales‐Jimenez et al. 2015), with the pinworms from *A. belzebuth* as part of the early divergent clades, followed by the lineage from *A. hybridus*, and, most recently, the clade of pinworms from *A. fusciceps and A. geoffroyi*. However, the lineages infecting *A. geoffroyi* subspecies (*A.g. vellerosus* distributed across southern Mexico, Belize, Guatemala, and Honduras; *A.g. frontatus* distributed in western and northern Nicaragua and northwestern Costa Rica—Rylands et al. 2006) were not recovered as sister clades in either species of pinworm. This was unexpected, given the close evolutionary relationship of their hosts. Likewise, the earliest divergent lineages seen in the different pinworm species were found in different *Ateles* hosts, suggesting processes of pinworm evolution other than co‐speciation.

Despite the considerable level of divergence observed within both *T. atelis* (5.6%) and *T. atelophora* (7.2%) across our sample of *Ateles* hosts, these values are below those typically seen between other *Trypanoxyuris* that are recognized as different species. For instance, the genetic divergence among *Trypanoxyuris* species known to parasitize different platyrrhine genera ranges from 9% to 12.5%, and that among species of pinworms that infect congeneric hosts ranges from 8.2% for *Trypanoxyuris* from *Alouatta* to 11.6% for *Trypanoxyuris* from *Aotus* monkeys (Solórzano‐García et al. 2016, 2024, 2020). Moreover, the lack of genetic variation in the 28S prevents considering any of the lineages recovered as distinct pinworm species, with the possible exception of lineage I (corresponding to *T. atelophora* from *A. belzebuth*), which showed the highest divergence values in both the ribosomal (28S) and mitochondrial (COI) genes (1.6% and 7.5%, respectively). Also, the higher genetic divergence observed within *T. atelophora* compared to *T. atelis* may indicate a more advanced stage of diversification, potentially associated with an earlier origin of *T. atelophora* lineage and, consequently, a longer time over which genetic differences have accumulated. Further sampling and detailed morphological and molecular analysis from male and female specimens are still required to further evaluate whether this clade warrants recognition as a new pinworm species.

Additionally, the genetic divergence among *Trypanoxyuris* lineages was greater than that among *Ateles* species. This suggests a faster rate of molecular evolution in the parasite compared to the host (which itself is unsurprising, given their much shorter generation time) even though the distinct pinworm lineages we found have not diverged sufficiently to be considered as separate species. A similar pattern has been observed in other parasites (i.e., Page et al. 1998; Paterson et al. 2000; Johnson and Clayton 2003; Chilton et al. 2004).

This apparent lack of complete divergence in the pinworms following speciation events within the *Ateles* radiation could be explained by two scenarios: (a) failure to speciate after host divergence, or (b) an ongoing speciation process in which the time since host diversification has not been enough for the parasite to accumulate sufficient differences. When closely related hosts acquire a parasite via ancestral inheritance, evolutionary inertia may prevent the parasite from completing the speciation process (Banks and Paterson 2005; Hugot et al. 2001). This phenomenon has been described in other host‐specific parasites such as lice infecting penguins and columbiform birds (Johnson et al. 2003; Paterson and Banks 2001), haemosporidian parasites of birds (Woodrow et al. 2023), and gastrointestinal nematodes of kangaroos (Chilton et al. 2004). Under this scenario, for example, recently diverged *A. geoffroyi* subspecies may have retained early divergent pinworm lineages (lineages E and H) (Figure 3), while parasite–host local adaptation continued in the other *Ateles* species, yielding moderate genetic differentiation between parasite lineages without speciation.

A failure to speciate interpretation requires that some level of gene flow persisted among pinworm populations after the initial diversification of their hosts. Although the current geographic distribution of the seven recognized species/subspecies of *Ateles* does not overlap, several species have adjacent ranges sharing borders (Collins and Dubach 2000), which may have historically facilitated contact among pinworm populations. Indeed, a recent mitogenomic study suggested that sympatric speciation may have played an important role in shaping current Platyrrhine diversity (Janiak et al. 2022), increasing the likelihood of parasite gene flow during host divergence.

Genetic configuration of parasite populations, as well as patterns of parasite diversification, can provide information on host dispersal and phylogeographical structure, making parasites useful tools for investigating the micro and macroevolutionary processes of their hosts (Nieberding and Olivieri 2007; Whiteman and Parker 2005). The phylogenetic associations we observed in both pinworm species could offer insights into the spider monkeys' diversification process and contribute to evaluating proposed hypotheses about their biogeographic history. For example, the relationships among *T. atelis* lineages we found here support the idea that the ancestor of *A. belzebuth* was part of an early divergent clade of spider monkeys that represented a northward expansion for some populations from southern Amazonia and then gave rise to *A. hybridus* and subsequently to the trans‐Andean species (*A. fusciceps* and *A. geoffroyi*) (Morales‐Jimenez et al. 2015). An alternative scenario places *A. hybridus* and *A. belzebuth*, together, as a sister group to the trans‐Andean clade (Morales‐Jimenez et al. 2015), which would predict sister relationships between their pinworm parasites. Our results suggest, however, that pinworms from *A. hybridus* are more closely related to those parasitizing the trans‐Andean hosts, in agreement with the first biogeographic proposal. Comprehensive phylogenetic analyses that incorporate pinworms from additional *Ateles* taxa, including subspecies, will be essential to better elucidate the biogeographic history of this parasite–host system.

Deciphering the evolutionary pathways that have shaped host–parasite associations is fundamental to understanding of ecological interactions, wildlife health, and biodiversity conservation. Our results demonstrate that pinworms infecting spider monkeys show a unique evolutionary history and genetic structure distinct from other *Trypanoxyuris*, highlighting the complexity and variability of parasite evolutionary dynamics. Filling the remaining gaps in our understanding on primate‐parasite associations will require collaborative studies across platyrrhine species and the routine integration of parasitological sampling into primate field research. As more information becomes available from pinworms infecting this and other primates, the coevolutionary framework of this remarkable system will continue to be refined, shedding light on the reciprocal influences that have shaped their diversification and biogeography.

## Author Contributions

**Brenda Solórzano‐García:** conceptualization, funding acquisition, writing – original draft, formal analysis, investigation. **Andrés Link Ospina:** conceptualization, investigation, writing – review and editing. **Anthony Di Fiore:** conceptualization, investigation, writing – review and editing. **Felipe Alfonso‐Cortes:** investigation. **Nathalia Fuentes:** investigation. **Gerardo Pérez‐Ponce de León:** investigation, writing – review and editing.

## Ethics Statement

This study was carried out entirely through noninvasive sampling under the permits MAE‐DNB‐CM‐2019‐0117 and CO48775, with no direct contact with individual animals; adhered to the American Society of Primatologists (ASP) Principles for the Ethical Treatment of Non‐Human Primates, and the ASP Code of Best Practices for Field Primatology.

## Conflicts of Interest

The authors declare no conflicts of interest.

## Supporting information


**Figure S1:** Haplotype network based on COI for *Trypanoxyuris atelis* and *T. atelophora*.**Table S1:** DNA sequences employed in the phylogenetic analysis of *Trypanoxyuris* species.**Table S2:** *Ateles* cytochrome b (cytb) data employed in genetic divergence analyses.

## Data Availability

All obtained sequences were deposited at GenBank. The data that support the findings of this study are openly available in GenBank at https://www.ncbi.nlm.nih.gov/genbank/, Reference Number PZ718919–42, PZ7122076–103.
