# Supplementary material for: Parasite–Host Associations in Pinworms (Trypanoxyuris spp.) From Spider Monkeys (Ateles spp.)
Source: Am J Primatol. 2026 Aug 27;88(8):e70203. doi: 10.1002/ajp.70203 (PMC13520131; doi:10.1002/ajp.70203)
Supplement: Supplementary file 1 [file AJP-88-e70203-s001.docx]

Supplementary material

**Parasite-host associations in pinworms (*Trypanoxyuris* spp.) from spider monkeys (*Ateles* spp.).**

Brenda Solórzano-García, Andrés Link Ospina, Anthony Di Fiore, Felipe Alfonso-Cortes, Nathalia Fuentes, Gerardo Pérez-Ponce de León.

**Table S1**. GenBank accession numbers of DNA sequences employed in the phylogenetic analysis of *Trypanoxyuris* species. Bold font indicates sequences obtained in this study. *Pinworm sequences obtained from hosts reared in zoos

| Species | Host | Country | GenBank accession numbers | |
| --- | --- | --- | --- | --- |
|  |  |  | COI | 28S |
| *Trypanoxyuris kemuimae* | *Alouatta seniculus* | Colombia |  | MN447700 – 01 |
| *Trypanoxyuris seunimii* | *Alouatta seniculus* | Colombia |  | MN447704 – 05 |
| *Trypanoxyuris pigrae* | *Alouatta pigra* | Mexico |  | KU285468 – 69 |
| *Trypanoxyuris minutus* | *Alouatta palliata* | Nicaragua | MH733439 | MH733400 |
|  |  | Mexico |  | KU285466 |
|  |  | Costa Rica | MH733420 | MH733397 |
|  | *Alouatta seniculus* | Colombia |  | MN447707 |
| *Trypanoxyuris kotudoi* | *Alouatta seniculus* | Colombia |  | MN447709 – 10 |
| *Trypanoxyuris multilabiatus* | *Alouatta palliata* | Costa Rica |  | MH733404 |
|  |  | Mexico |  | KU285470 |
|  |  | Nicaragua |  | MH733405 |
| *Trypanoxyuris atelis* | *Ateles geoffroyi* | Mexico | MF379120  MF379129  MF379096  MF379112 | KU285474 KP266350 |
|  |  | Costa Rica | MH733422 – 25 | MH733406 – 07 |
|  |  | Japan* | AB626875 |  |
|  | ***Ateles fusciceps*** | **Ecuador** | **PZ722091 – 97** | **PZ718932 – 38** |
|  | ***Ateles hybridus*** | **Colombia** | **PZ722076 – 81** | **PZ718919 – 24** |
|  | ***Ateles belzebuth*** | **Ecuador** | **PZ722082 – 90** | **PZ718925 – 31** |
|  |  | Japan* | AB626876 |  |
|  | *Lagothrix lagotricha* | Japan* | AB626877 |  |
| *Trypanoxyuris atelophora* | *Ateles geoffroyi* | Mexico | KP266379  MH733426  KP266373 | KP266376  KP266371 |
|  |  | Costa Rica | MH733428 – 30 | MH733409 – 10 |
|  | ***Ateles hybridus*** | **Colombia** | **PZ722098 – 99** | **PZ718939 – 40** |
|  | ***Ateles belzebuth*** | **Ecuador** | **PZ722100 – 03** | **PZ718941 – 42** |
| *Trypanoxyuris microon* | *Aotus griseimembra* | Colombia | OR506588 | OR509779 |
| *Trypanoxyuris interlabiata* | *Aotus griseimembra* | Colombia |  | OR509772 |
| *Enterobius vermicularis* | *Homo sapiens* | Japan | AB626868 |  |

**Table S2.** Mitochondrial cytochrome b (cytb) data employed in genetic divergence analyses

| Species | GenBank accession number |
| --- | --- |
| *Ateles belzebuth* | OM328920 |
| *Ateles belzebuth* | KJ452772 |
| *Ateles chamek* | OM328957 |
| *Ateles chamek* | OM329059 |
| *Ateles fusciceps* | KJ452768 |
| *Ateles fusciceps* | KR902385 |
| *Ateles fusciceps* | KJ452769 |
| *Ateles fusciceps* | KR902370 |
| *Ateles geoffroyi frontatus* | HM222708 |
| *Ateles geoffroyi vellerosus* | KR902388 |
| *Ateles geoffroyi vellerosus* | AY065900 |
| *Ateles geoffroyi yucatanensis* | AY065902 |
| *Ateles hybridus* | KR902383 |
| *Ateles hybridus* | KJ452766 |
| *Ateles marginatus* | KJ507210 |
| *Ateles marginatus* | NC_064192 |
| *Ateles paniscus* | OM328958 |
| *Ateles paniscus* | KJ452765 |
| *Ateles paniscus* | NC064191 |

**Figure S1.** Minimum Spanning haplotype network based on COI for *Trypanoxyuris atelis* and *T. atelophora* from different *Ateles* hosts. Haplotype frequency is represented by size of the circle. Small lines indicate mutational steps between haplotypes. GEO: *A. geoffroyi vellerosus*, FRO: *A. geoffroyi frontatus*, FUS: *A. fusciceps*, HYB: *A. hybridus*, BEL: *A. belzebuth*.
